# Reduced childbirth rates in multiple sclerosis from the prodromal phase: Evidence from a population-based cohort study

**DOI:** 10.1177/13524585251315077

**Published:** 2025-02-17

**Authors:** Cecilia Smith Simonsen, Heidi Øyen Flemmen, Line Broch, Kamilla Brekke, Harald Myklebust, Pål Berg-Hansen, Cathrine Brunborg, Elisabeth Gulowsen Celius

**Affiliations:** Department of Neurology, Vestre Viken Hospital Trust, Drammen, Norway; Department of Neurology, Hospital Telemark HF, Skien, Norway; Department of Neurology, Vestre Viken Hospital Trust, Drammen, Norway; Department of Neurology, Oslo University Hospital, Oslo, Norway; Department of Neurology, Vestfold Hospital, Tønsberg, Norway; Institute of Clinical Medicine, University of Oslo, Oslo, Norway; Department of Neurology, Vestre Viken Hospital Trust, Drammen, Norway; Institute of Clinical Medicine, University of Oslo, Oslo, Norway; Department of Neurology, Oslo University Hospital, Oslo, Norway; Research Support Services, Oslo Centre for Biostatistics and Epidemiology, Oslo University Hospital, Oslo, Norway; Department of Neurology, Oslo University Hospital, Oslo, Norway; Institute of Clinical Medicine, University of Oslo, Oslo, Norway

**Keywords:** Multiple sclerosis, birth rate, prodrome, marital status, women with MS

## Abstract

**Background:**

The age of multiple sclerosis (MS) onset coincides with fertile age, and both improved prognosis and treatment may influence birth rates in people with MS (pwMS).

**Objectives:**

To investigate birth rates over time in pwMS compared with controls.

**Methods:**

This cohort study included pwMS from three hospitals in the southeast of Norway. Clinical data were collected prospectively. Statistics Norway provided year of live births and marital status in pwMS and controls matched for age, sex, and place of residence at age 16.

**Results:**

We included 1599 pwMS (1118 women with MS (wMS) and 481 men with MS (mMS)) and 23,855 controls. The mean number of live births was 1.5 (standard deviation (SD) 1.2) for pwMS versus 1.8 (SD 1.2) for controls (*p* < 0.001). Birth rates in wMS declined significantly starting 3 years before onset, with 4.5% giving birth versus 8.4% of controls 2 years before onset (*p* = 0.046). Birth rates were also lower 1 year after onset (*p* = 0.002). mMS showed a dip 2 years before onset (*p* = 0.002), but otherwise had rates similar to controls. There were no differences in marital status.

**Conclusion:**

wMS have reduced rates of childbirth compared with controls. This is significant already in the prodromal phase.

## Introduction

Multiple sclerosis (MS) is a chronic disease with a female predominance and onset in young adulthood, often coinciding with the period of establishing a family and having children.^1^ Several studies have indicated a prodromal phase with vague symptoms predating the onset of classical disease, increased number of health care contacts and prescriptions up 10 years before diagnosis.^2,3^ In addition, an increase in neurofilament level years before clinical onset has been demonstrated.^4^

Over the past three decades, several disease-modifying therapies (DMTs) have become available, coinciding with a milder disease course and improved prognosis.^5^ Many DMTs are contraindicated during pregnancy, and treatment choices may influence family planning and contribute to a lower birth rate among women with MS (wMS).^6^ There are limited data on infertility among wMS. One claims study found a higher rate of infertility diagnoses and a lower rate of infertility treatments in wMS. The birth rate following infertility treatments in wMS was similar to healthy controls.^7^ During pregnancy, the disease in wMS usually remains stable, but there is an increase in disease activity postpartum in untreated wMS, though this is less pronounced in wMS on DMTs.^8,9^ There are few studies and conflicting evidence regarding the effect of pregnancies on the disease in the long term.^10,11^

Two studies have shown a reduced birth rate among wMS,^12,13^ but to our knowledge, birth rates have not been studied in men with MS (mMS). One study from the United States showed a decline in birth rates in healthy women over the years but an increase among wMS in the same period. The authors concluded that this was a consequence of newer and more effective DMTs.^14^

The aim of this study was to investigate the birth rate over time in a Norwegian population-based cohort of people with MS (pwMS) and compare this to population controls matched for age, sex, and place of residence at age 16.

## Methods

This is a cohort study with matched controls.

All living pwMS in the two south-eastern Norwegian counties Buskerud and Telemark, as well as a large part of the Oslo MS population, were invited to participate in a broader study focusing on MS and socioeconomic status. Of the 2512 living pwMS in these counties, 1599 responded (64% response rate). Those who provided informed consent were enrolled in the BOT-MS registry. The non-responders were slightly older (53.9 14 vs 51.8 13, *p* < 0.001) with a longer disease duration (21 years from onset (interquartile range 13–30) versus 16 years (interquartile range 8–25), *p* < 0.001).^15^ This registry offers comprehensive insights into disease onset, diagnosis, and disease characteristics, as described in detail in a previous publication.^5^ Clinical data were retrieved from hospital records and collected for this study and was concluded on January 1, 2018. Each Norwegian citizen possesses a unique identity number, facilitating precise person identification across various health districts and databases. Statistics Norway provided data on marital status and live births annually in all pwMS as of January 1, 2018. Statistics Norway has kept records of birth statistics since 1876. In addition, the same information was provided for up to 15 controls per pwMS, matched based on sex, age, and county of residence at age 16. Residency at age 16 was chosen, as this age typically reflects the social and environmental context in which pwMS spent their formative years, thus providing a common baseline and reducing potential confounding related to early-life socioeconomic status and environmental factors, which are more stable at this age. Socioeconomic status within Norway’s counties is relatively homogeneous compared with many other countries due to the country’s equitable distribution of resources. To look at temporal changes in age of first birth and births before and after onset/diagnosis, we stratified the groups into five groups dependent on pwMS’ decade born: 1941–1950, 1951–1960, 1961–1970, 1971–1980, and 1981–1990. We did not include pwMS born before 1941 or after 1990 as only 21 and 4 pwMS had given birth in these age groups. For the category of marital status, the term “unmarried” includes both those who are single and cohabitants. We refer to number of births among men and women as birth rates.

### Statistics

We used IBM SPSS Statistics 29.0 (IBM Corp., Armonk, NY, USA) for data analysis. Between groups, differences in continuous variables were tested with Student’s t-test for normally distributed data. The chi-square test for contingency tables was used to detect associations between categorical variables. One-way analysis of variance (ANOVA) was used to compare means across the five subgroups. Mean number of children born stratified by year of birth for cases (wMS and mMS) and controls were plotted in a diagram and fitted with a linear trendline. Linear regression using number of live births as a continuous outcome variable was used to detect a trend in live births. Linear regression analysis was used to show trends in births stratified by pwMS/control’s year of birth. Linear mixed effect models regression was used to investigate if longitudinal changes in live births differed between cases (pwMS) and controls. Years from onset or diagnosis, pwMS/control, and the interaction between time from onset or diagnosis*pwMS/control intercept were included as fixed effects in the models. All models included random intercept and slope. We modeled time by using a piecewise linear spline with a knot at the time of onset or diagnosis. All p-values were two-sided, and a 5% significance level was used.

### Ethical considerations

All participants provided written informed consent. The Regional Ethics Committee (REK 2015/670) and the Data Protection Officer approved the study under the condition that strict privacy concerns were respected. Consequently, data will not be made publicly available and specific requests regarding data sharing should be directed to the corresponding author.

## Results

### Demographics

We included 1599 pwMS (cases), of which 1118 women and 481 men, and 23,855 matched controls. The pwMS had a mean 1.5 (standard deviation (SD) 1.2) live births and the controls had 1.8 (SD 1.2) live births (*p* < 0.001).

**Figure 1.**
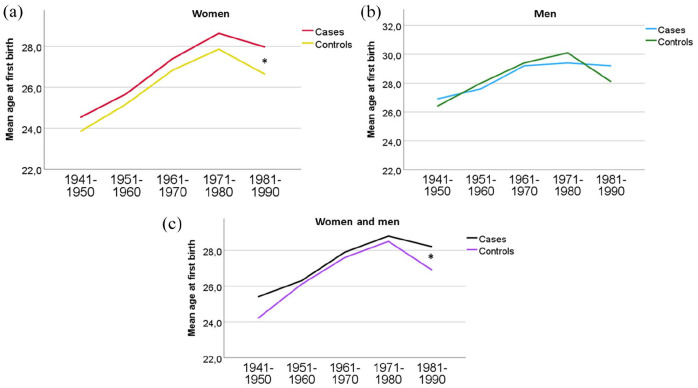
Mean age of first birth by decade pwMS was born in (a) both sexes, (b) mMS, and (c) wMS. *Significant difference between cases and controls in all pwMS born between 1981 and 1990 (28.2 (SD 4.5) vs 26.9 (SD 4.3) years, *p* = 0.001) and in wMS born between 1981 and 1990 (28.0 (SD 4.5) vs 26.6 (SD 4.4), *p* = 0.007).

Mean age of first birth was significantly higher in wMS, though the absolute difference was relatively small (26.8 (SD 5.4) vs 26.1 (SD 5.3), *p* < 0.001). There was no significant difference in the mean age at first birth among mMS (28.3 (SD 5.2) vs 28.5 (SD 5.7), *p* = 0.589). For wMS, there were no significant differences in mean age of first birth by decade between 1940 and 1980, but between 1981 and 1990, the wMS were 1.3 (0.5) years older than controls (28.0 (SD 4.5) vs 26.9 (SD 4.3), *p* = 0.007). There were no significant differences in any decade between mMS and controls (Figure 1).

We did not find a significant difference in marital status between cases and controls. Table 1 shows demographics for cases and controls, and Table 2 shows disease characteristics of the pwMS. Supplementary Table 1 shows disease demographics by decade of birth.

**Table 1.** PwMS and controls demographics.

	All		*p*	Women		*p*	Men		
	pwMS	Controls		wMS	Controls		mMS	Controls	*p*
*N* =	1599	23,855		1118	16,671		481	7184	
Age, mean years (SD)	52.4 (13.5)	52.4 (13.5)	0.455	51.8 (13.6)	51.8 (13.6)	0.458	54.0 (13.0)	54.0 (13.0)	0.483
Live births (SD)	1.5 (1.1)	1.8 ^(1–2)^	*<0.001*	1.5 (1.2)	1.8 (1.2)	*<0.001*	1.5 (1.2)	1.8 (1.3)	*<0.001*
Mean age of first birth	27.3 (5.4)	26.8 (5.5)	*0.004*	26.8 (5.4)	26.3 (5.3)	*<0.001*	28.3 (5.2)	28.5 (5.7)	0.589
Marital status 2018									
Unmarried, %	31	31	0.678	31	31	0.934	31	32	
Married/widowed, %	52	52		51	51		54	54	0.537
Separated/divorced, %	18	17		19	18		16	14	
Marital status age 30									
Unmarried, %	50	48		48	45	0.307	54	42	
Married/widowed %	45	46	0.560	46	48		55	5	0.700
Separated/divorced, %	6	5		6	6		55	4	
Marital status age 40									
Unmarried, %	25	26		24	24	0.792	26	31	
Married/widowed, %	62	61	0.398	62	61		62	59	0.169
Separated/divorced, %	13	13		14	14		12	11	
Marital status age 50									
Unmarried, %	17	18		16	17	0.832	18	21	
Married/widowed, %	62	62	0.482	62	62		63	63	0.452
Separated/divorced, %	21	20		22	22		19	17	
Marital status age 60									
Unmarried, %	10	9		12	9	0.111	7	11	
Married/widowed, %	69	70	0.765	65	69		74	71	0.*326*
Separated/divorced, %	21	21		23	23		19	18	
Marital status age 70									
Unmarried, %	5	7		6	6	0.995	2	8	
Married/widowed, %	77	75	0.626	74	73		82	78	0.*339*
Separated/divorced, %	19	18		20	20		16	14	

SD: standard deviation; pwMS: people with MS; wMS: women with MS; mMS: men with MS.

Significant values are in italic.

**Table 2.** PwMS disease characteristics.

	pwMS	wMS	mMS
Age onset, mean (SD)	34.4 (10.5)	33.8 (10.6)	35.7 (10.1)
Age diagnosis, mean (SD)	39.5 (11.3)	38.9 (11.4)	40.9 (10.9)
Time from onset to diagnosis, mean (SD)	5.1 (6.9)	5.1 (7.0)	5.2 (6.8)
Progressive disease at onset, %	8.9	6.6	10.0
Ever treated DMT, %	58.5	59.3	56.5
Ever treated high efficacy DMT, %	28.4	28.1	29.2

SD: standard deviation; pwMS: people with MS; wMS: women with MS; mMS: men with MS; DMT: disease-modifying therapy.

### Live births by parental year of birth

Table 3 shows the number of births by decade of parental birth. wMS have consistently given less birth compared with controls over the decades, while there were few differences between mMS and controls.

**Table 3.** Number of births by decade of parental birth.

	PwMS			wMS			mMS		
	Cases (SD)	Control (SD)	*p*	Cases (SD)	Controls (SD)	*P*	Cases (SD)	Controls (SD)	*p*
1941–1950	1.8 (1.1)	2.2 (1.2)	*<0.001*	1.7 (1.1)	2.2 (1.1)	*<0.001*	2.0 (1.1)	2.2 (1.3)	0.239
1951–1960	1.8 (1.1)	2.0 (1.2)	*0.002*	1.8 (1.1)	2.0 (1.1)	*0.004*	1.8 (1.2)	2.0 (1.2)	0.170
1961–1970	1.7 (1.2)	1.9 (1.2)	*<0.001*	1.7 (1.2)	2.0 (1.2)	*0.001*	1.7 (1.3)	1.8 (1.3)	0.133
1971–1980	1.4 (1.1)	1.8 (1.2)	*<0.001*	1.6 (1.1)	1.9 (1.1)	*<0.001*	1.1 (1.0)	1.6 (1.2)	*<0.001*
1981–1990	0.8 (0.9)	1.1 (1.1)	*<0.001*	0.8 (0.9)	1.2 (1.1)	*<0.001*	0.8 (0.9)	0.8 (1.0)	0.947

SD: standard deviation.

Significant values are in italic.

**Figure 2.**
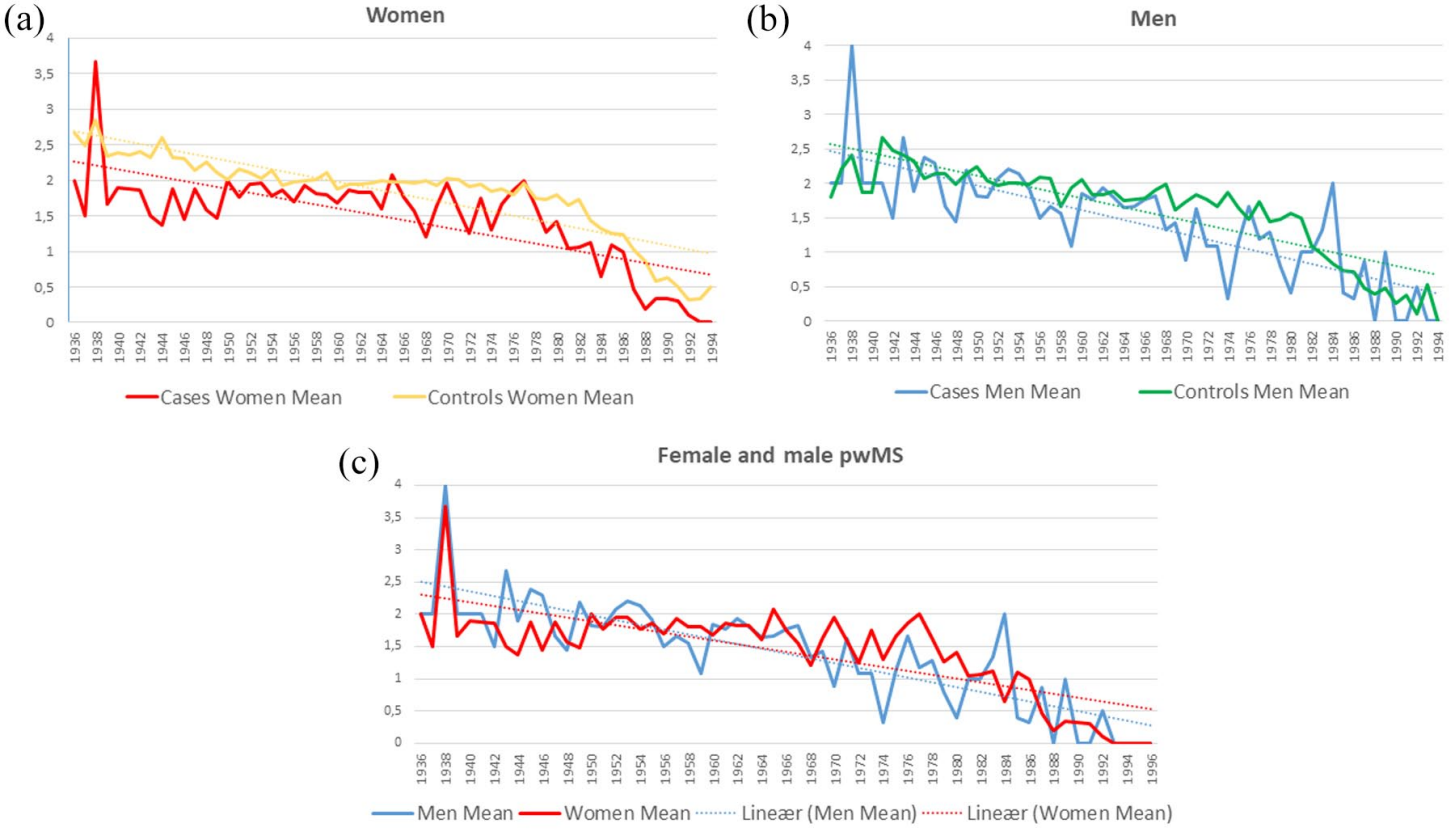
**(a)** Mean number of children born stratified by year of birth of mother for cases and controls in women. Blue dotted linear trendline formula for cases: *y* = −0.0274*x* + 2.2899. Orange dotted linear trendline formula for controls *y* = −0.0297*x* + 2.714. **(b)** Mean number of children born stratified by year of birth of father for cases and controls. Blue dotted linear trendline formula for cases: *y* = −0.0358*x* + 2.5109. Green dotted linear trendline formula *y* = −0.0327*x* + 2.5987. **(c)** Mean number of children born stratified by year of birth for male (blue) and female (red) pwMS.

Figure 2(a) and (b) shows the mean number of births stratified by year of parental births in men and women. wMS had significantly fewer children compared with men in all five decades. There is a significant difference between the number of children in cases and controls over time. The difference between cases and controls over time decreases slightly in women, while it increases slightly in men. The difference between wMS and female controls is still larger than their male counterparts throughout the study period, but the slope difference between mMS and wMS was not significantly different (0.00001 (95% confidence interval (CI) −0.00007 to 0.00010), *p* = 0.761; see Figure 2(c) and Table 3).

### Live births before and after onset and diagnosis

Compared with controls, significantly fewer wMS gave birth 3 years and 2 years before *onset*, as well as 1 year and 2 years after onset. Significantly fewer male pwMS compared with controls had children 2 years before onset, but, otherwise, there were no significant differences. There were no differences in the year before or the year of onset in either women or men (Table 4).

**Table 4.** Regression analysis showing trends in births, stratified by pwMS/control’s year of birth.

		*Β*	95% CI	*P*
All	Trend birth cases	−0.033	−0.037 to −0.028	*<0.001*
	Trend birth controls	−0.033	−0.036 to −0.029	*<0.001*
	Slope difference over time	−0.0001	−0.0002 to 0.0001	*<0.001*
Women	Trend birth cases	−0.029	−0.036 to −0.023	*<0.001*
	Trend birth controls	−0.032	−0.036 to −0.028	*<0.001*
	Slope difference over time	−0.0002	−0.0002 to −0.0001	*<0.001*
Men	Trend birth cases	−0.036	−0.043 to −0.030	*<0.001*
	Trend birth controls	−0.034	−0.039 to −0.029	*<0.001*
	Slope difference over time	−0.0001	−0.0002 to −0.0001	*0.014*

CI: confidence interval.

Significant values are in italic.

**Figure 3.**
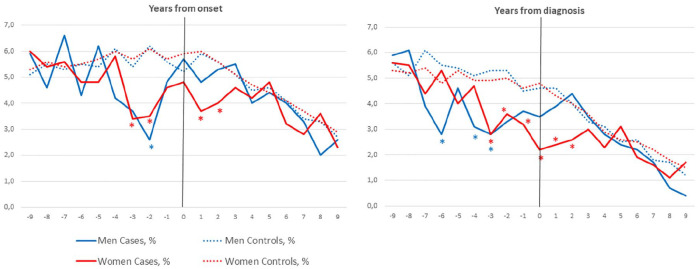
Percentage of male and female pwMS and controls with live births relative to onset and diagnosis (year 0). *Significant difference between cases and controls.

Compared with controls, significantly fewer wMS gave birth in the 3 years leading up to *diagnosis*, the year of diagnosis, as well as the 2 years after diagnosis. For mMS, there was a significant reduction in live births compared with controls in years 6, 4, 3, and 1 leading up to diagnosis, but not after diagnosis. Figure 3 shows the live births over time in female (red) and male (blue) pwMS, as well as controls, in relation to year of onset and diagnosis. Table 5 shows the percentage of cases and controls with live births at year of onset and diagnosis and the 9 years before and after onset/diagnosis.

**Table 5.** The percentage of cases and controls with live births at year of onset and diagnosis and the 9 years before and after onset/diagnosis.

	Onset						Diagnosis					
	Men			Women			Men			Women		
	Cases, *n* = 455 *n* =, (%)	Controls, *n* = 6809 *n* =, (%)	*p*	Cases, *n* = 1052 *n* =, (%)	Controls, *n* = 15,696 *n* =, (%)	*p*	Cases, *n* = 459 *n* =, (%)	Controls, *n* = 6869 *n* =, (%)	*p*	Cases, *n* = 1081 *n* =, (%)	Controls, *n* = 16,131 *n* =, (%)	*p*
9 years before	27 (5.9)	349 (5.1)	*0.451*	63 (6.0)	843 (5.3)	*0.346*	27 (5.9)	384 (5.6)	*0.792*	61 (5.6)	851 (5.3)	*0.602*
8 years before	21 (4.6)	375 (5.5)	*0.417*	57 (5.4)	881 (5.6)	*0.790*	28 (6.1)	349 (5.1)	*0.338*	59 (5.5)	873 (5.2)	*0.700*
7 years before	30 (6.6)	359 (5.3)	*0.226*	59 (5.6)	853 (5.4)	*0.810*	18 (3.9)	422 (6.1)	*0.052*	48 (4.4)	587 (0.4)	*0.177*
6 years before	19 (4.3)	377 (5.5)	*0.216*	50 (4.8)	870 (5.5)	*0.276*	13 (2.8)	376 (5.5)	*0.015*	57 (5.3)	772 (4.8)	*0.469*
5 years before	28 (6.2)	365 (5.4)	*0.469*	50 (4.8)	897 (5.7)	*0.191*	21 (4.6)	371 (5.4)	*0.446*	43 (4.0)	861 (5.3)	*0.052*
4 years before	19 (4.2)	413 (6.1)	*0.099*	61 (5.8)	936 (6.0)	*0.827*	14 (3.1)	351 (5.1)	*0.050*	51 (4.7)	478 (8.9)	*0.805*
3 years before	17 (3.7)	367 (5.4)	*0.127*	36 (3.4)	893 (5.7)	*0.002*	13 (2.8)	361 (5.3)	*0.022*	30 (2.8)	796 (4.9)	*0.001*
2 years before	12 (2.6)	420 (6.2)	*0.002*	37 (3.5)	963 (6.1)	*<0.001*	15 (3.3)	366 (5.3)	*0.054*	39 (3.6)	813 (5.0)	*0.036*
1 year before	22 (4.8)	381 (5.6)	*0.493*	48 (4.6)	892 (5.7)	*0.126*	17 (3.7)	308 (4.5)	*0.432*	35 (3.2)	744 (4.6)	*0.035*
**Year 0**	**26 (5.7)**	**352 (5.2)**	***0.613***	**50 (4.8)**	**920 (5.9)**	***0.136***	**16 (3.5)**	**310 (4.6)**	***0.301***	**24 (2.2)**	**778 (4.8)**	***<0.001***
1 year after	22 (4.8)	399 (5.9)	*0.365*	39 (3.7)	938 (6.0)	*0.002*	18 (3.9)	316 (4.6)	*0.500*	26 (2.4)	699 (4.3)	*0.002*
2 year after	24 (5.3)	381 (5.6)	*0.773*	42 (4.0)	876 (5.6)	*0.028*	20 (4.4)	273 (4.0)	*0.685*	28 (2.6)	646 (4.0)	*0.020*
3 year after	25 (5.5)	344 (5.1)	*0.677*	48 (4.6)	799 (5.1)	*0.450*	16 (3.5)	229 (3.3)	*0.861*	32 (3.0)	573 (3.6)	*0.306*
4 year after	18 (4.0)	308 (4.5)	*0.571*	44 (4.2)	740 (4.7)	*0.429*	13 (2.8)	213 (3.1)	*0.747*	25 (2.3)	473 (2.9)	*0.239*
5 year after	20 (4.4)	310 (4.6)	*0.876*	51 (4.8)	691 (4.4)	*0.497*	11 (2.4)	175 (2.5)	*0.842*	34 (3.1)	427 (2.6)	*0.326*
6 year after	18 (4.0)	280 (4.1)	*0.871*	34 (3.2)	646 (4.1)	*0.160*	10 (2.2)	180 (2.6)	*0.564*	21 (1.9)	397 (2.5)	*0.284*
7 year after	15 (3.3)	234 (3.4)	*0.874*	29 (2.8)	579 (3.7)	*0.118*	8 (1.7)	124 (1.8)	*0.923*	17 (1.6)	356 (2.2)	*0.166*
8 year after	9 (2.0)	227 (3.3)	*0.114*	38 (3.6)	523 (3.3)	*0.625*	3 (0.7)	116 (1.7)	*0.089*	12 (1.1)	284 (1.8)	*0.111*
9 year after	12 (2.6)	185 (2.7)	*0.919*	24 (2.3)	456 (2.9)	*0.240*	2 (0.4)	83 (1.2)	*0.134*	18 (1.7)	236 (1.5)	*0.134*

Significant values are highlighted in italics, with year 0 (the year of onset and diagnosis) in bold.

In the regression analyses, there was a significant reduction in trend in live births over time in the 9 years before onset (−0.246 (95% CI −0.423 to −0.069), *p* = 0.009) and 9 years before diagnosis (0.384 (95% CI −0.419 to 0.147), *p* < 0.001) for wMS compared with controls. There was no significant reduction in trend in live births for mMS in the 9 years before onset or diagnosis.

When stratifying according to pwMS’ decade born, we found significantly fewer births in the year before onset in wMS born between 1971 and 1980 (4.5% vs 8.4%, *p* = 0.046) and 1981 and 1990 (1.9% vs 6.4%, *p* = 0.020). We also found significantly fewer births in the year of onset in wMS born between 1981 and 1990 (1.2% vs 7.0%, *p* = 0.004). We did not find similar trends in mMS (see supplementary Table 2).

## Discussion

Our study shows a lower birth rate among pwMS compared with the general population. There is also a reduction in birth rate over time for both sexes, which parallels the reduction in the general population worldwide.^16^ The observed decrease in total birth rate aligns with findings from two recent studies from Denmark and Italy, showing reduced birth rates among wMS.^12,13^ However, these results contrast with a U.S. study, which showed an increase in birth rate among wMS.^14^ The difference is most likely due to selection bias in the U.S. study. The Scandinavian studies comprise pwMS in geographically well-defined areas, and in our study, the controls are population-based and matched for age, sex, and place of residence at age 16. Thus, we aimed at comparing pwMS with socioeconomically comparable controls.

The distribution of marital status of our pwMS does not differ from that of the control group. This finding contrasts with a Danish study that reported more older pwMS living alone^13^ and a Swedish study that found a higher divorce rate among mMS, but not wMS.^17^ Again, we believe this is due to the composition of the control group, with less impact by socio-economic status in the current study.

Another important finding in this study is the more pronounced reduction in birth rate among pwMS, and in particular female pwMS, in the last years before both symptom onset and diagnosis of MS. This adds a new dimension to the disease prodrome. Previous studies have shown increased numbers of healthcare visits due to anxiety, depression, pain, and cognitive problems in the years before MS onset.^2,3^ Although the prodrome affects both sexes, there may be some differences in symptoms.^3,18^ A Canadian population-based cohort study showed a 14% reduction in pregnancy and childbirth-related issues, and an increase in prescriptions of oral contraceptive in the 5 years leading up to the first demyelinating claim.^19^ The Canadian study utilized two definitions for the index date: the first demyelinating disease-related diagnostic code in the health administrative cohort and symptom onset in the smaller clinical cohort. In our study, we have precise data for both the onset of symptoms and diagnosis (not the first claim), allowing us to delineate temporal changes in birth rates with greater accuracy. We found significant differences in birth rates in the year before and the year of onset for women born between 1971 and 1990. In addition, wMS born between 1981 and 1990 were, on average, older at the time of their first birth. With each decade, more women were started on DMT, and 46% of women born between 1981 and 1990 were started on high efficacy therapy. One hypothesis is that women are waiting for a stable disease before getting pregnant. Improved access to healthcare and abortion services in modern times might provide women with greater autonomy when their perceived health is poor. The observed dip in birth rates in the years preceding MS onset in our study may reflect trends in conception rather than just live births. This could be influenced by early symptoms or prodromal experiences that affect health and well-being, leading individuals to delay or avoid conception. This may also explain why fewer differences in birth rates are observed in the year of symptom onset, as conception decisions would likely have been influenced in the preceding year(s). Our findings should be replicated in a larger population to further explore the differences in the MS prodrome between younger and older women.

Male pwMS regain a birth rate similar to controls after diagnosis, while women trail behind healthy controls both before and after diagnosis. Treatment with DMTs could explain some of the differences in birth rates between females and males after diagnosis, as many of the DMTs used are contraindicated in pregnancy. However, given the relatively stable birth rate ratio between pwMS and controls over time, including periods before DMTs were available, it is clear that DMTs do not fully explain the trends. Surveys have shown that pwMS are more likely to have no children and many report that the diagnosis impacts their family planning decision-making.^20–22^ Proactive discussion with women and their families is essential and requires careful consideration.^6^ DMTs were introduced almost 30 years ago, but the use was limited in the first years, and there was a hesitancy in prescribing drugs to wMS planning a pregnancy. In recent years, the change in diagnostic criteria^23^ allows for an earlier diagnosis, and at the same time, the concept of early high-efficacy therapy has been applied, both of which may affect birth rates, especially in wMS. These changes are too recent to be fully captured by this study, and further studies are warranted. The mean age of first-time pregnancies has increased and is now 30 years in Norwegian women.^24^ Thus, the lower birth rates after 1990 (Figure 1(a) and (b)) should be interpreted with caution, as these pwMS are still young and might have more children in the next few years.

The strength of our study is the large population-based cohort and the use of validated registry data, avoiding recall bias. We used controls matched for age, sex, and municipality of residency at age 16, to avoid differences caused by socioeconomic status. Socioeconomic status influences birth rates^16^ but is largely maintained across generations. This study does not investigate sexual dysfunction and fertility in pwMS, but our data do not indicate either reduced fertility or sexual dysfunction as a major cause of reduced birth rate in pwMS. The data from Statistics Norway does not differentiate between single and cohabitants, so this entire group is referred to as “unmarried.” There may be differences between pwMS and controls in the number of single people in this group. Finally, healthy survivor bias could be a factor in this study, as including only those who were alive and willing to consent might skew the sample toward healthier individuals or those with milder disease courses. However, the inclusion of individuals with more advanced MS would likely have revealed more pronounced differences.

## Conclusion

Our study shows a reduced rate of childbirths in females with MS compared with healthy controls. In particular, there is a dip in birth rates among women in the 3 years leading up to MS symptom onset, most likely due to the MS prodrome. Females, but not males, display a reduced rate of childbirths after diagnosis compared with controls, and for both sexes, there is a gradual reduction in birth rate over decades, similar to the general population.

## Supplemental Material

sj-docx-1-msj-10.1177_13524585251315077 – Supplemental material for Reduced childbirth rates in multiple sclerosis from the prodromal phase: Evidence from a population-based cohort study Supplemental material, sj-docx-1-msj-10.1177_13524585251315077 for Reduced childbirth rates in multiple sclerosis from the prodromal phase: Evidence from a population-based cohort study by Cecilia Smith Simonsen, Heidi Øyen Flemmen, Line Broch, Kamilla Brekke, Harald Myklebust, Pål Berg-Hansen, Cathrine Brunborg and Elisabeth Gulowsen Celius in Multiple Sclerosis Journal

sj-docx-2-msj-10.1177_13524585251315077 – Supplemental material for Reduced childbirth rates in multiple sclerosis from the prodromal phase: Evidence from a population-based cohort study Supplemental material, sj-docx-2-msj-10.1177_13524585251315077 for Reduced childbirth rates in multiple sclerosis from the prodromal phase: Evidence from a population-based cohort study by Cecilia Smith Simonsen, Heidi Øyen Flemmen, Line Broch, Kamilla Brekke, Harald Myklebust, Pål Berg-Hansen, Cathrine Brunborg and Elisabeth Gulowsen Celius in Multiple Sclerosis Journal
