# Supplementary material for: Reduced childbirth rates in multiple sclerosis from the prodromal phase: Evidence from a population-based cohort study
Source: Mult Scler. 2025 Feb 17;31(4):398–407. doi: 10.1177/13524585251315077 (PMC11956373; doi:10.1177/13524585251315077)
Supplement: sj-docx-1-msj-10.1177_13524585251315077 – Supplemental material for Reduced childbirth rates in multiple sclerosis from the prodromal phase: Evidence from a population-based cohort study [file sj-docx-1-msj-10.1177_13524585251315077.docx]

**Supplementary table 1**: Disease demographics by decade of birth

|  |  | **1941-1950** | **1951-1960** | **1961-1970** | **1971-1980** | **1981-1990** | ***p*** |
| --- | --- | --- | --- | --- | --- | --- | --- |
| **All pwMS** | Age onset, mean (SD) | 41,0 (11.8) | 38,3 (10.9) | 35,4 (8.9) | 31,4 (6.7) | 25,5 (4.8) | <0.001 |
|  | Age diagnosis, mean (SD) | 50,3 (11.0) | 45.0 (9.8) | 40,4 (7.7) | 34,2 (6.1) | 27,1 (4.3) | <0.001 |
|  | Time from onset to diagnosis, mean (SD) | 9.5 (9.7) | 6,9 (7.7) | 5,1 (6.0) | 2,9 (3.9) | 1,6 (2.6) | <0.001 |
|  | Progressive disease at onset, % | 24 | 14 | 10 | 1 | 1 | <0.001 |
|  | Ever treated DMT, % | 15 | 39 | 69 | 81 | 85 | <0.001 |
|  | Ever treated high efficacy DMT, % | 4 | 15 | 33 | 38 | 46 | <0.001 |
|  |  |  |  |  |  |  |  |
| **wMS** | Age onset, mean (SD) | 41.2 (12.0) | 37.7 (11.0) | 35.2 (9.2) | 31.1 (7.0) | 25.1 (4.7) | <0.001 |
|  | Age diagnosis, mean (SD) | 50.4 (10.7) | 44.9 (9.9) | 40.0 (7.7) | 33.9 (6.4) | 26.8 (4.3) | <0.001 |
|  | Time from onset to diagnosis, mean (SD) | 9.4 (9.6) | 7.6 (8.2) | 5.0 (6.2) | 3.0 (3.9) | 1.6 (2.6) | <0.001 |
|  | Progressive disease at onset, % | 22 | 10 | 3 | 1 | 2 | <0.001 |
|  | Ever treated DMT, % | 13 | 40 | 71 | 79 | 83 | <0.001 |
|  | Ever treated high efficacy DMT, % | 4 | 13 | 33 | 38 | 44 | <0.001 |
|  |  |  |  |  |  |  |  |
| **mMS** | Age onset, mean (SD) | 40.8 (11.4) | 39.7 (10.8) | 35.0 (6.1) | 32.0 (6.0) | 26.8 (5.0) | <0.001 |
|  | Age diagnosis, mean (SD) | 50.2 (11.5) | 45.2 (9.6) | 41.3 (7.1) | 37.8 (5.2) | 28.3 (5.0) | <0.001 |
|  | Time from onset to diagnosis, mean (SD) | 9.6 (10.0) | 5.6 (6.5) | 5.3 (5.7) | 2.7 (3.9) | 1.6 (2.4) | <0.001 |
|  | Progressive disease at onset, % | 28 | 23 | 13 | 1 | 1 | <0.001 |
|  | Ever treated DMT, % | 18 | 38 | 63 | 86 | 92 | <0.001 |
|  | Ever treated high efficacy DMT, % | 4 | 19 | 34 | 39 | 46 | <0.001 |
|  |  |  |  |  |  |  |  |

*SD standard deviation, pwMS people with MS, wMS women with MS, mMS men with MS, DMT disease modifying therapy*
