# Supplementary material for: Reduced childbirth rates in multiple sclerosis from the prodromal phase: Evidence from a population-based cohort study
Source: Mult Scler. 2025 Feb 17;31(4):398–407. doi: 10.1177/13524585251315077 (PMC11956373; doi:10.1177/13524585251315077)
Supplement: sj-docx-2-msj-10.1177_13524585251315077 – Supplemental material for Reduced childbirth rates in multiple sclerosis from the prodromal phase: Evidence from a population-based cohort study [file sj-docx-2-msj-10.1177_13524585251315077.docx]

**Supplementary table 2**: Percentage of cases and controls with live births at year of onset and the five years before and after, stratified by decade born

|  |  |  | **Onset** | | | | | | | | | | | | | | | | | | | | | |
| --- | --- | --- | --- | --- | --- | --- | --- | --- | --- | --- | --- | --- | --- | --- | --- | --- | --- | --- | --- | --- | --- | --- | --- | --- |
|  |  |  | **5 years before** | ***p*** | **4 years before** | ***p*** | **3 years before** | **p** | **2 years before** | **p** | **1 year before** | **p** | **Year 0** | **p** | **1 year after** | **p** | **2 year after** | **p** | **3 year after** | **p** | **4 year after** | **p** | **5 year after** | **p** |
| **All** | **1941-1950** | **Cases** | 3,6 % | 0,304 | 6,7 % | 0,477 | 2,7 % | 0,117 | 2,2 % | 0,083 | 1,3 % | 0,084 | 5,4 % | 0,206 | 3,6 % | 0,874 | 1,3 % | 0,047 | 4,9 % | 0,051 | 2,2 % | 0,824 | 1,3 % | 0,207 |
|  |  | **Controls** | 5,1 % |  | 5,6 % |  | 5,0 % |  | 4,8 % |  | 3,5 % |  | 3,7 % |  | 3,8 % |  | 4,0 % |  | 2,7 % |  | 2,5 % |  | 2,8 % |  |
|  | **1951-1960** | **Cases** | 5,8 % | 0,586 | 4,9 % | 0,611 | 3,5 % | 0,162 | 4,0 % | 0,396 | 4,6 % | 0,706 | 5,8 % | 0,355 | 2,9 % | 0,279 | 2,9 % | 0,266 | 2,9 % | 0,447 | 2,3 % | 0,344 | 3,2 % | 0,583 |
|  |  | **Controls** | 5,1 % |  | 5,5 % |  | 5,2 % |  | 5,1 % |  | 4,2 % |  | 4,7 % |  | 4,1 % |  | 4,1 % |  | 3,7 % |  | 3,2 % |  | 3,7 % |  |
|  | **1961-1970** | **Cases** | 6,8 % | 0,891 | 5,0 % | 0,183 | 3,8 % | 0,075 | 3,5 % | *0,010* | 7,8 % | 0,224 | 5,0 % | 0,591 | 4,3 % | 0,167 | 4,8 % | 0,584 | 5,5 % | 0,807 | 5,0 % | 0,748 | 4,8 % | 0,680 |
|  |  | **Controls** | 6,6 % |  | 6,7 % |  | 5,9 % |  | 6,8 % |  | 6,2 % |  | 5,6 % |  | 5,9 % |  | 5,4 % |  | 5,2 % |  | 4,7 % |  | 4,3 % |  |
|  | **1971-1980** | **Cases** | 6,6 % | 0,570 | 7,2 % | 0,680 | 5,5 % | 0,269 | 3,1 % | 0,004 | 4,8 % | *0,037* | 7,2 % | 0,540 | 4,8 % | *0,029* | 6,6 % | 0,465 | 6,6 % | 0,700 | 7,6 % | 0,701 | 7,6 % | 0,404 |
|  |  | **Controls** | 7,5 % |  | 7,9 % |  | 7,2 % |  | 7,7 % |  | 8,3 % |  | 8,3 % |  | 8,5 % |  | 7,7 % |  | 7,2 % |  | 7,0 % |  | 6,3 % |  |
|  | **1981-1990** | **Cases** | 2,0 % | 0,227 | 3,4 % | 0,574 | 2,0 % | 0,057 | 3,4 % | 0,054 | 2,9 % | 0,051 | 1,5 % | *0,003* | 5,4 % | 0,117 | 7,3 % | 0,778 | 5,4 % | 0,290 | 3,4 % | 0,070 | 7,8 % | 0,167 |
|  |  | **Controls** | 3,5 % |  | 4,2 % |  | 4,8 % |  | 6,9 % |  | 6,3 % |  | 6,7 % |  | 8,5 % |  | 7,9 % |  | 7,3 % |  | 6,6 % |  | 5,5 % |  |
| **Women** | **1941-1950** | **Cases** | 2,1 % | 0,088 | 6,2 % | 0,726 | 2,8 % | 0,238 | 3,4 % | 0,519 | 2,1 % | 0,372 | 5,5 % | 0,389 | 3,4 % | 0,822 | 0,0 % | *0,028* | 3,4 % | 0,652 | 1,4 % | 0,714 | 0,7 % | 0,249 |
|  |  | **Controls** | 5,3 % |  | 5,5 % |  | 4,9 % |  | 4,6 % |  | 3,4 % |  | 4,0 % |  | 3,8 % |  | 3,2 % |  | 2,8 % |  | 1,8 % |  | 2,1 % |  |
|  | **1951-1960** | **Cases** | 5,6 % | 0,808 | 6,0 % | 0,670 | 3,4 % | 0,186 | 5,2 % | 0,878 | 3,4 % | 0,681 | 6,0 % | 0,436 | 3,9 % | 0,967 | 3,4 % | 0,529 | 2,6 % | 0,485 | 2,1 % | 0,362 | 2,6 % | 0,358 |
|  |  | **Controls** | 5,2 % |  | 5,4 % |  | 5,4 % |  | 4,9 % |  | 4,0 % |  | 4,9 % |  | 3,9 % |  | 4,3 % |  | 3,4 % |  | 3,2 % |  | 3,7 % |  |
|  | **1961-1970** | **Cases** | 5,4 % | 0,367 | 5,1 % | 0,326 | 3,2 % | 0,062 | 2,2 % | *0,003* | 9,0 % | 0,056 | 4,0 % | 0,260 | 3,6 % | 0,149 | 4,3 % | 0,438 | 5,4 % | 0,664 | 5,8 % | 0,345 | 4,7 % | 0,579 |
|  |  | **Controls** | 6,8 % |  | 6,6 % |  | 5,9 % |  | 6,6 % |  | 6,1 % |  | 5,6 % |  | 5,7 % |  | 5,4 % |  | 4,8 % |  | 4,5 % |  | 4,0 % |  |
|  | **1971-1980** | **Cases** | 7,4 % | 0,966 | 8,9 % | 0,787 | 5,4 % | 0,306 | 4,0 % | 0,048 | 4,5 % | *0,046* | 7,4 % | 0,595 | 4,0 % | *0,030* | 5,4 % | 0,258 | 7,4 % | 0,823 | 7,9 % | 0,652 | 8,4 % | 0,239 |
|  |  | **Controls** | 7,5 % |  | 8,4 % |  | 7,4 % |  | 7,7 % |  | 8,4 % |  | 8,5 % |  | 8,2 % |  | 7,6 % |  | 7,0 % |  | 7,1 % |  | 6,3 % |  |
|  | **1981-1990** | **Cases** | 2,5 % | 0,405 | 3,7 % | 0,707 | 2,5 % | 0,164 | 3,7 % | 0,095 | 1,9 % | *0,020* | 1,2 % | *0,004* | 4,3 % | *0,040* | 6,8 % | 0,613 | 4,3 % | 0,092 | 3,1 % | 0,039 | 8,6 % | 0,225 |
|  |  | **Controls** | 3,7 % |  | 4,3 % |  | 4,9 % |  | 7,1 % |  | 6,4 % |  | 7,0 % |  | 9,0 % |  | 7,9 % |  | 8,0 % |  | 7,4 % |  | 6,2 % |  |
| **Men** | **1941-1950** | **Cases** | 6,4 % | 0,545 | 7,7 % | 0,474 | 2,6 % | 0,301 | 0,0 % | *0,042* | 0,0 % | 0,089 | 5,1 % | 0,319 | 3,8 % | 0,969 | 3,8 % | 0,557 | 7,7 % | 0,007 | 3,8 % | 0,969 | 2,6 % | 0,522 |
|  |  | **Controls** | 4,9 % |  | 5,7 % |  | 5,2 % |  | 5,0 % |  | 3,6 % |  | 3,1 % |  | 3,8 % |  | 5,4 % |  | 2,5 % |  | 3,8 % |  | 4,0 % |  |
|  | **1951-1960** | **Cases** | 6,1 % | 0,542 | 2,6 % | 0,143 | 3,5 % | 0,597 | 1,8 % | 0,093 | 7,0 % | 0,247 | 5,3 % | 0,617 | 0,9 % | 0,072 | 1,8 % | 0,281 | 3,5 % | 0,735 | 2,6 % | 0,728 | 4,4 % | 0,730 |
|  |  | **Controls** | 4,9 % |  | 5,9 % |  | 4,6 % |  | 5,3 % |  | 4,6 % |  | 4,3 % |  | 4,3 % |  | 3,7 % |  | 4,2 % |  | 3,2 % |  | 3,7 % |  |
|  | **1961-1970** | **Cases** | 9,8 % | 0,098 | 4,9 % | 0,356 | 4,9 % | 0,685 | 6,5 % | 0,782 | 4,9 % | 0,500 | 7,3 % | 0,492 | 5,7 % | 0,719 | 5,7 % | 0,859 | 5,7 % | 0,861 | 3,3 % | 0,411 | 4,9 % | 0,954 |
|  |  | **Controls** | 6,0 % |  | 7,1 % |  | 5,8 % |  | 7,2 % |  | 6,4 % |  | 5,8 % |  | 6,5 % |  | 5,3 % |  | 6,1 % |  | 4,9 % |  | 5,0 % |  |
|  | **1971-1980** | **Cases** | 4,5 % | 0,327 | 3,4 % | 0,207 | 5,7 % | 0,650 | 1,1 % | 0,023 | 5,7 % | 0,458 | 6,8 % | 0,761 | 6,8 % | 0,484 | 9,1 % | 0,720 | 4,5 % | 0,305 | 6,8 % | 0,993 | 5,7 % | 0,786 |
|  |  | **Controls** | 7,3 % |  | 6,9 % |  | 6,9 % |  | 7,6 % |  | 7,9 % |  | 7,7 % |  | 9,0 % |  | 8,0 % |  | 7,5 % |  | 6,8 % |  | 6,4 % |  |
|  | **1981-1990** | **Cases** | 0,0 % | 0,267 | 2,3 % | 0,606 | 0,0 % | 0,141 | 2,3 % | 0,328 | 7,0 % | 0,805 | 2,3 % | 0,377 | 9,3 % | 0,478 | 9,3 % | 0,714 | 9,3 % | 0,216 | 4,7 % | 0,757 | 4,7 % | 0,482 |
|  |  | **Controls** | 2,8 % |  | 3,9 % |  | 4,8 % |  | 5,9 % |  | 6,0 % |  | 5,4 % |  | 6,5 % |  | 7,8 % |  | 5,0 % |  | 3,7 % |  | 2,8 % |  |
